# MR Imaging of Hepatocellular Adenomas and Differential Diagnosis Dilemma

**DOI:** 10.1155/2013/374170

**Published:** 2013-03-27

**Authors:** Luigi Grazioli, Lucio Olivetti, Giancarlo Mazza, Maria Pia Bondioni

**Affiliations:** ^1^Department of Radiology, Spedali Civili, 25100 Brescia, Italy; ^2^Department of Radiology, Istituti Ospitalieri di Cremona, 26100 Cremona, Italy; ^3^Department of Radiology, University of Brescia, Spedali Civili, 25100 Brescia, Italy

## Abstract

Hepatocellular adenomas (HCAs) are currently categorized into distinct genetic and pathologic subtypes as follows: inflammatory hepatocellular adenoma, hepatocyte-nuclear-factor-1-alpha (HNF-1**α**-mutated) hepatocellular adenoma, and **β**-catenin-mutated hepatocellular adenomas; the fourth, defined as unclassified subtype, encompasses HCAs without any genetic abnormalities. This classification has accepted management implications due to different risks of haemorrhage and malignant transformation of the four subtypes. Imaging guided biopsy and/or surgical resection very important in obtaining definitive characterization; nevertheless, MRI with intra-extravascular and hepatobiliary (dual phase) agents, is an important tool not only in differential subtypes definition but even in surveillance with early identification of complications and discovery of some signs of HCA malignant degeneration. Inflammation, abnormal rich vascularisation, peliotic areas, and abundant fatty infiltration are pathologic findings differently present in the HCA subtypes and they may be detected by multiparametric MRI approach. Lesion enlargement and heterogeneity of signal intensity and of contrast enhancement are signs to be considered in malignant transformation. The purpose of this paper is to present the state of the art of MRI in the diagnosis of HCA and subtype characterization, with particular regard to morphologic and functional information available with dual phase contrast agents, and to discuss differential diagnosis with the most common benign and malignant lesions mimicking HCAs.

## 1. Introduction

Hepatocellular adenoma (HCA) is a rare benign tumour (incidence of 1/1,000,000) that is mainly found in women of child-bearing age (second most frequent hepatocellular tumor in young women after focal nodular hyperplasia). There is evidence that HCA is strongly related to current and recent (first generation, high dose) oral contraceptives (OC) use. Recent, low-dose OCs appear less strongly, at all, related to HCA [1]. Sometimes tumour regression has been noted after discontinuation of OC. Non-OC-related causes of HCA include familial insulin-dependent diabetes, Fanconi anaemia, glycogen storage diseases, and hormonal stimulation from other sources, for instance, anabolic steroid use by body builders, gynaecological tumours, or pregnancy [2–6]. 

Small HCA is generally asymptomatic. Right upper abdominal quadrant fullness or discomfort is present in 40% of cases due to mass effect. Typical clinical manifestation is spontaneous rupture or haemorrhage leading to acute abdominal pain with possible progression to hypotension and even death. HCA rarely undergoes malignant transformation to hepatocellular carcinoma (HCC). Laboratory tests for liver function are usually normal. Because of the different therapeutic options HCA must be distinguished from other hypervascular lesions (HCC, fibrolamellar HCC, focal nodular hyperplasia (FNH), and metastases) that may occur in young adults without cirrhosis. 

Steroid related adenoma is typically detected when it reaches about 5 cm in diameter but the size could be 6–30 cm (large and multiple lesions are more prone to spontaneous haemorrhage). In 75% of cases the lesion involves the subcapsular region of right lobe of liver, in 10% it is intraparenchymal or pedunculated. 

HCA is usually solitary (70–80%) and multiple in 20–30%. A so-called adenomatosis is present in subjects observed to have more than 10 lesions: this entity is independent of gender or hormone therapy [7, 8].

Treatment criteria include number and size of lesions, presence of symptoms, and surgical risk. The possible role for elective surgery mainly depends upon the risk of complications, but data concerning its actual incidence are presently lacking. In fact, both selection and spectrum bias negatively affect the retrospective surgical case series that represent most of the available literature on this topic. Moreover, concerning the two other common indications for surgery, represented by the uncertainty of diagnosis and the presence of symptoms related to tumour size, the role of surgery can be challenged. In fact, the continuous improvement in diagnostic techniques, particularly magnetic resonance, has the consequence that one can restrict surgical resection to very few participants for which the procedure has not only a curative target, but also the need for a precise pathologic definition. Also the role of surgery for symptomatic tumors has been claimed only by small uncontrolled case series [9]. However, as a general rule, if HCA is <5 cm in size, discontinuation of OC and radiological follow-up are acceptable; if the lesion is >5 cm or near hepatic surface, due to the recognized risk of rupture and haemorrhage, surgical resection is the treatment of choice. Pregnancy should be avoided due to increased risk of rupture.

According to recent studies HCAs are currently categorized in four distinct genetic and pathologic subtypes as follows: inflammatory hepatocellular adenomas, hepatocyte-nuclear-factor-1-alpha- (HNF-1*α*-mutated) hepatocellular adenomas, and *β*-catenin-mutated hepatocellular adenomas. Finally HCAs without any genetic abnormalities are categorized in the unclassified subtype [10–13]. Although this classification is new and not yet widely accepted, it has definitive management implications. Image-guided biopsy or surgical resection with histopathologic and immunohistochemical analysis is necessary for complete characterization of HCAs but MR imaging plays an important role in diagnosis and subtype characterization as well as identification of complications and surveillance [14, 15].

The purpose of this paper is to present MR imaging characteristics of specific subtypes of HCAs by using dual phase contrast agents and to discuss differential diagnosis with the most common benign and malignant lesions mimicking HCAs.

## 2. Intra- and Extravascular and Hepatobiliary Agents

Gadobenate dimeglumine (Gd-BOPTA, Multihance, Bracco) and gadoxetic acid (Gd-GD-EOB-DTPA, Primovist, Bayer) differ from purely extracellular gadolinium agents as they combine the properties of conventional nonspecific gadolinium agents with those of an agent targeted specifically to hepatocytes: for this reason they are also called *hepatospecific* contrast agents. With these contrast media it is possible to perform dynamic phase imaging as performed with conventional gadolinium agents and delayed-phase imaging as performed with mangafodipir trisodium (Teslascan, GE Healthcare). Using Gd-BOPTA arterial, portal venous, and equilibrium phase images are readily obtainable using identical sequences, the same contrast speed injection to those employed with nonspecific gadolinium agents. In a different way, considering the very fast hepatocyte intake of Gd-EOB-DTPA, the equilibrium phase is spurious because of the overlap between interstitial and hepatocyte times. Indeed, hepatocyte phase after Gd-EOB-DTPA starts after 3 minutes of contrast medium injection. There is an overlap between equilibrium and early hepatocyte phases; therefore, equilibrium appearance after Gd-EOB-DTPA is spurious. 

Unlike the conventional agents, approximately 3–5% and 50%, respectively, of the injected doses of Gd-BOPTA and Gd-EOB-DTPA are taken up by functioning hepatocytes and ultimately excreted via the biliary system. The result of the hepatocytic uptake is that the normal liver parenchyma shows strong enhancement on delayed T1w images that is maximal approximately 2-3 hours after Gd-BOPTA injection and 20 minutes after Gd-EOB-DTPA administration. A second feature unique to Gd-BOPTA and Gd-EOB-DTPA is that the contrast-effective moiety of these agents interacts weakly and transiently with serum albumin. This interaction shows the tumbling rate of the Gd-BOPTA and Gd-EOB-DTPA chelates and results in longer rotation correlation times in shell water protons for Gd-BOPTA and Gd-EOB-DTPA compared to generic gadolinium agents that do not interact with serum albumin. This in turn results in a T1 relaxivity in human plasma at 37°C at 1.5 T that is approximately that of conventional gadolinium agents (r_1_ 7, 3 and 6, 7 for Gd-EOB-DTPA and Gd-BOPTA, resp., in comparison to 4, 2–4, 6 with conventional Gd-chelates) [16]. Not only does this increased relaxivity permit lower overall doses to be used to acquire the same information of dynamic as available with conventional agents at a standard dose, but it also facilitates the improved performance of the Gd-BOPTA and Gd-EOB-DTPA for both intra- and extrahepatic vascular imaging.

A principal advantage of the selective uptake by functioning hepatocytes is that the normal tissue enhances while tumors of nonhepatocytic origin such as metastases and cholangiocarcinoma, as well as nonfunctioning tumor that are unable to uptake Gd-BOPTA and Gd-EOB-DTPA, remain unenhanced, thereby increasing the liver contrast-noise ratio (CNR) and hence the ability to detect lesion.

MR imaging is typically performed, being Gd-BOPTA and Gd-EOB-DTPA T1 relaxation time contrast agents, with 2D or 3D Gradient-Echo sequences while the use of fat saturation has been shown to raise CNR on dynamic and delayed hepatobiliary phase imaging.

Clinical studies and routine clinical practice have shown that the dynamic phase imaging is particularly important for lesion characterization while delayed phase imaging in the hepatobiliary phase increases the sensitivity of MRI for liver detection. However, delayed phase imaging also contributes to improving the characterization of lesions, particularly when results of unenhanced and dynamic sequences are equivocal or when atypical enhancement patterns are noted on dynamic imaging [17–21].

## 3. Characterization of HCAs Subtypes with MRI

### 3.1. Inflammatory HCAs

Inflammatory HCA is the most common subtype and accounts for about 30%–50% of all hepatocellular adenomas. These tumors are mainly seen in women, in association with obesity, hepatic steatosis, diabetes mellitus, glycogenesis (in particular, type I glycogen storage diseases), and alcohol abuse. More than 90% of women have a history of contraceptive use [22]. Patients with inflammatory HCA may present with signs of chronic anemia and/or “systemic inflammatory syndrome,” characterized by fever, leukocytosis, and elevated serum levels of C-reactive protein [23]. Inflammatory HCAs are associated with a definitive increased risk of bleeding (>30%) and a risk of malignant transformation (5–10%) [10, 12]. They comprise a prototype example of tumours induced by hepato-biliary inflammation: more than 40% of genes overexpressed in inflammatory HCAs are associated with inflammation and immune response. Around 10% of inflammatory HCAs may also show mutation involving *β* catenin gene.

Histologically, inflammatory HCAs are characterized by significant sinusoidal dilatation, polymorphous inflammatory infiltrates, areas of peliosis, and thickened tortuous arteries. Prominent ductal reaction represents the distinct histological feature. Steatosis within nodule is variable and less extensive compared with HNF-1*α*-mutated HCAs [15, 24]. 

**Figure 1:**
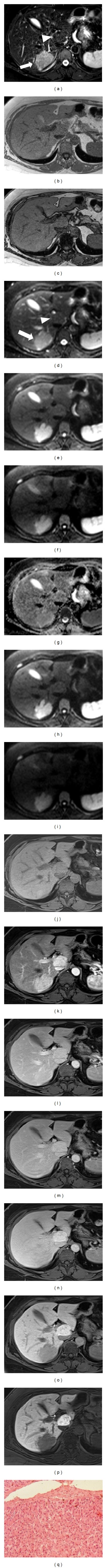
Inflammatory adenoma and focal nodular hyperplasia. (a) Axial T2w fat-suppressed image. In Segment (S) VII homogeneous well-delineated hyperintense adenoma (arrow), proven by biopsy. In SI, note a second isointense lesion, focal nodular hyperplasia (arrowhead). (b-c) T1w in- and out-phased images. Both lesions are mainly isointense. (d–i) DWI sequences. Adenoma (arrow in d) is constantly hyperintense. Conversely FNH (arrowhead in d) is isointense. ADC map (i) both lesions are is slightly hyperintense. (j–n) In dynamic evaluation after Gd-EOB-DTPA administration adenoma shows intense enhancement in arterial phase (k) with washout in portal (l) and equilibrium (m) phases; conversely, FNH shows progressive increase of the signal. (o-p) In hepatobiliary phases after 10′ (o) and 20′ (p), adenoma is hypointense relative to the adjacent liver parenchyma and FHN is hyperintense. (o) Histology shows hepatocytes arranged in plates that are two to three cells thick separated by sinusoids.

**Figure 2:**
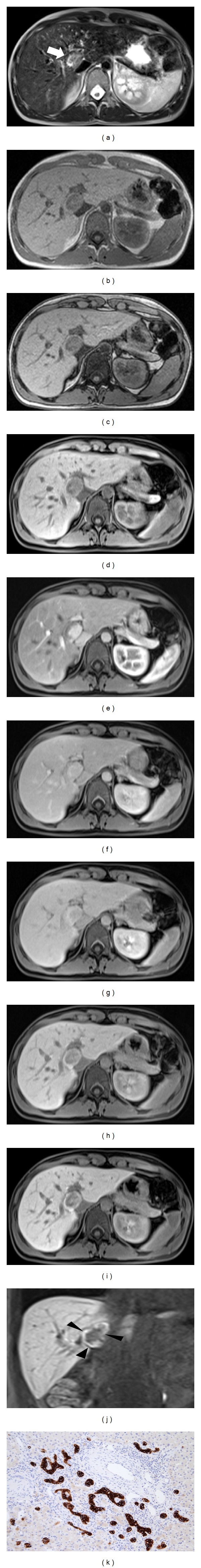
Inflammatory adenoma with “atoll sign.” (a) In S IX well-delineated slightly hyperintense lesion with hyperintense peripheral rim (arrow) in axial T2w image. (b-c) T1w in- and out-phased images. The mass is hypointense relative to the hepatic parenchyma with isointense rim. (d–h) In dynamic evaluation after Gd-EOB-DTPA administration the lesion shows marked central enhancement in arterial phase (e) with slight wash out in portal (f) and more evident equilibrium (g) phases. After 5′ (h) the nodule is mainly hypointense. Conversely, the peripheral component of the nodule demonstrates progressive enhancement over time. (i-j) In hepatobiliary phases after 20′, axial (i) and coronal (j), the lesion is heterogeneously hypointense in the central portion and hyperintense at the periphery (arrowheads). Final diagnosis was obtained by biopsy. Histology demonstrates ductal reaction at the periphery of the lesion (k).

**Figure 3:**
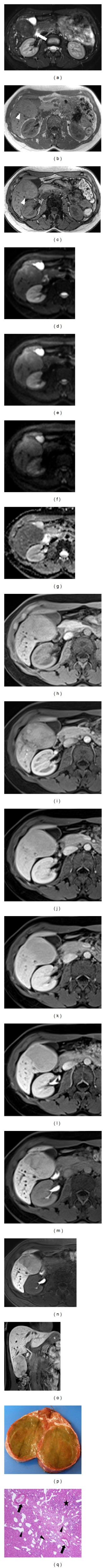
Atypical inflammatory adenoma. (a) In S VI homogeneous well-delineated isointense lesion in axial T2w fat-suppressed image (arrow). (b-c) T1w in- and out-phased images. The mass is isointense relative to the hepatic parenchyma. Note large vessels at the periphery of the lesion (arrowheads). (d–g) DWI sequences. The lesion does not show any increase of signal intensity from 50 (d) to 400 (e) and to 800 (f) *b* values. IN ADC map (g) the nodule shows isointense signal. (h–l) In dynamic evaluation after Gd-EOB-DTPA administration the mass shows discrete enhancement in arterial phase (i) with slightly wash out in portal (j) and equilibrium (k) phases. After 5′ (l) the nodule is more hypointense. (m-n) In hepatobiliary phases after 10′ (m) and 20′ (n), the lesion is definitively heterogeneously hypointense relative to the adjacent liver parenchyma with exophytic growth. (o) Cut section shows large capsulated homogeneous mass. (p) Microscopically, significant sinusoidal dilatation (arrowheads), polymorphous inflammatory infiltrates (star), areas of peliosis, and thickened tortuous arteries (arrow) are demonstrated.

On plain MR imaging inflammatory HCA is often hyperintense on T2w images and hypointense on T1w sequence, frequently with heterogeneous signal intensity. Hyper- and hypointensity on T2w and T1w images, respectively, correspond mainly to areas of sinusoidal dilatation and inflammatory infiltrates. Focal areas of fat may be seen as hypointense areas on T1 out-phased images due to signal drop. Inflammatory HCA may appear as a hypervascular mass with persistent enhancement during dynamic evaluation and may show a variable uptake in the hepatobiliary phase specially at the periphery (Figure 1). Sometimes because of sinusoidal dilatation, inflammatory component and ductal reaction, in the hepato-biliary phase image areas of hypointensity in adenomas, mainly in the periphery, may be seen [25, 26]. Marked T2 hyperintensity associated with delayed persistent enhancement has a sensitivity of as much as 85% and a specificity of 87% for the diagnosis of inflammatory HCA. Peripheral hyper-intensity (*atoll sign*) reflects the abnormal ductal reaction with alterated biliary excretion (Figure 2) [13, 14]. In a small percent of cases, inflammatory HCA may appear isointense on T2 and T1w images with discrete enhancement in arterial phase and quite rapid wash out (Figure 3). 

### 3.2. HNF-1*α*-Mutated HCAs

HNF-1*α*-mutated HCA is the second most common type; it constitutes about 30–35% of all HCAs and arises because of biallelic inactivation of transcription factor 1 gene located in chromosome twelve. This kind of adenoma is nearly exclusively seen in women, except for rare HCA with germline HFN-1*α* mutations which can be also observed in men. Hepatocyte nuclear 1*α* mutation may be somatic or less frequently germline in origin. The final outcome of this mutation is the production of nonfunctioning HNF-1*α* protein which promotes lipogenesis by suppression of gluconeogenesis, activation of glycolysis, and promotion of fatty acid biosynthesis. The reduction of fatty acid binding protein 1 leads to faulty transport of fatty acids and to intracellular deposition of fat. Indeed, HNF-1*α*-mutated HCA is characterized by diffuse intralesional steatosis. HNF-1*α* mutation may be the primary inciting event that results in the accumulation of estrogen metabolites that unconditionally stimulate hepatocyte proliferation [27, 28].

On MR examination, HNF-1*α*-mutated HCA often shows heterogeneous hypointensity areas on T1 out-phased sequences with significant signal drops on out-phased in comparison with in-phased sequences, corresponding to fatty deposition. Hyperintensity on T1 in-phased and out-phased images signal drop may correspond to glycogen component or less commonly haemorrhage. On T2w images the lesion tends to appear as iso- or hypointense nodule without significant restriction on DWI. This is true in uncomplicated adenomas; conversely, complicated adenomas or adenomas containing different tissues may show restriction. ADC maps may appear aspecific, with positive or negative values between 0.9 and 1.3 [29, 30].

**Figure 4:**
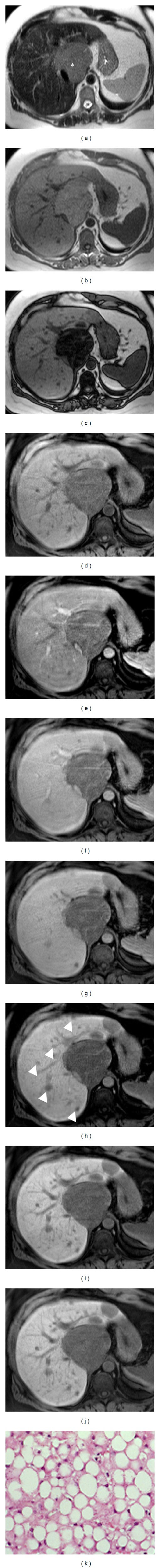
HNF-1*α*-mutated adenomas. (a) Axial T2w images without fat suppression. In S I and S II multiple slightly homogeneous hyperintense lesions (asterisk indicates the largest nodule biopsied). (b-c) T1w in- and out-phased images. Due to significant intralesional presence of fat tissue, the nodules show signal drop with variable degree in c. (d–h) In dynamic evaluation after Gd-EOB-DTPA administration the nodules show poor enhancement. In (h), after 5′ the nodules are more hypointense; other lesions may be detected in both hepatic lobes (arrowheads). (i-j) In hepatobiliary phases after 10′ (i) and 20′ (j), all adenomas are hypointense relative to adjacent liver parenchyma. (k) Histology confirms presence of fat-rich hepatocytes.

On dynamic evaluation after Gd-BOPTA and Gd-EOB-DTPA, HNF-1*α*-mutated HCA appears hypervascular with variable degrees, but usually less evident than inflammatory adenoma. On portal venous and equilibrium phases the lesion tends to be hypointense; on hepatobiliary phase the mass appears hypointense in almost all cases with homogeneous appearance (Figure 4). 

Significant signal drop in T1 out-phased imaging for predictive HNF-1*α*-mutated HCA is reported to be 85%, 100%, 100%, 94% of sensitivity, specificity, predictive positive, and negative predictive value, respectively [14].

### 3.3. *β*-Catenin-Mutated HCAs

*β*-Catenin-mutated HCAs constitute about 10–15% of all HCAs; they originate from sustained activation of *β*-catenin because of mutations involving the CTNNB1 gene (catenin *β*1). These tumors primarily involve patients with glycogen storage disease and on androgen treatment and have a greater propensity to undergo malignant transformation to HCCs. *β*-Catenin plays a major role in hepatocyte development, differentiation, proliferation, and regeneration. Activation of *β*-catenin in normal hepatocyte is usually transient and is regulated by its rapid degradation. Excessive nuclear accumulation and sustained activation of *β*-catenin may result from mutation in *β*-catenin itself or from mutation involving cytoplasmic degradation complex. Excessive *β*-catenin activity results in autonomous growth of hepatocyte and accelerates HCA formation [22, 27, 31]. 

Although *β*-catenin mutation is implicated in malignant transformation of HCA, only 20–30% of malignant HCAs show *β*-catenin mutation. Glycogen storage disease is an additional independent risk for malignant HCA transformation. Up to 75% of patients, glycogen storage disease may develop HCA [32]. 

**Figure 5:**
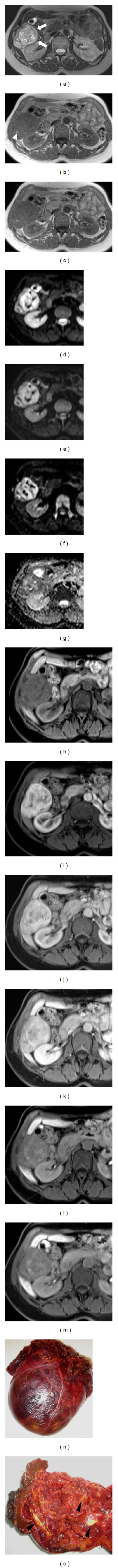
*β*-catenin-mutated adenoma. (a) In S VI heterogeneous well delineated hyperintense lesion (arrows). (b-c) T1w in- and out-phased images. The mass is slightly hypointense relative to hepatic parenchyma with focal signal drop due to intralesional fatty infiltration (arrowhead). (d–g) DWI sequences. The lesion increases signal intensity from 50 (d) to 400 (e) and to 800 (f) *b* values. In ADC map (g) the nodule shows heterogeneous signal. (h–l) In dynamic evaluation after Gd-EOB-DTPA administration the mass shows intense heterogeneous enhancement in arterial phase (i) without evident wash out in portal (j) and (k) equilibrium phases. (l-m) In hepatobiliary phases after 10′ (l) and 20′ (m), the lesions are heterogeneous hypointense relative to the adjacent liver parenchyma. (n-o) Gross specimen confirms capsulated heterogeneous mass with fatty component (arrowheads).

On MR imaging *β*-catenin-mutated HCA appears as homogeneous or heterogeneous hypervascular mass with persistent or nonpersistent enhancement during the delayed-phase images. Signal intensity on T2 and on T1 precontrast sequences is variable but mainly heterogeneously hyper-and hypointense, respectively. Malignant transformation simulates HCC on imaging and does not show peculiar findings (Figure 5) [14].

### 3.4. Unclassified HCAs

**Figure 6:**
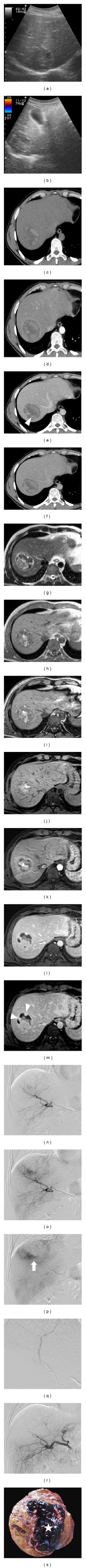
Bleeding adenoma in young man with sudden and acute upper abdominal pain. (a-b) Ultrasound, B-mode (a) and color-Doppler (b) imaging show heterogeneous lesion without significant vascularization. (c–f) CT confirms heterogeneous hyperdense bleeding mass with persistent vascularized tissue in the lower portion of the lesion (arrowheads). (g) Axial T2w image shows mixed heterogenous bleeding capsulated mass (arrows) in S VIII. (h-i) T1w in- and out-phased images confirm intralesional hemorrhage. (j–m) MR dynamic study shows vascularized tissue in the lower portion of the lesion (arrowheads). (n–p) DSA before embolization. Note hypervascular tissue in the lower part of the lesion (arrow). (q-r) DSA after embolization, complete devascularization of the nodule. (s) Cut section of the resected lesion shows large hemorrhagic component (star).

Approximately 10% of all HCAs are without specific genetic and/or pathologic abnormalities. Frequently, the presence of haemorrhage may be one of the reasons that justify the unclassified categorization of the lesion (Figure 6). No specific MR imaging patterns have yet been proposed to identity unclassified HCAs also because imaging experience is very limited [10, 22].

No immunohistochemical analyses were performed to compare with MRI features, allowing to assert the diagnosis of HCA subtype.

## 4. Differential Diagnosis Dilemma

Some benign and malignant lesions may simulate HCA. The differential diagnosis depends on clinical and MRI findings. 

### 4.1. Focal Nodular Hyperplasia

Focal nodular hyperplasia is the second most common benign hepatic tumor (8% of primary hepatic tumors at autopsy). FNH represents an hyperplastic response to a localized vascular abnormality; consequently it is not a true benign tumor but a benign congenital hamartomatous malformation.

FNH is predominantly found in the same group of patients as well as HCA: female patients (M : F = 1 : 8), usually in 3rd-4th decades of life, with history of OC consumption. Multiple FNHs are found in 10–20% of cases and association with hemangioma occurs in 5–10% of cases.

Patients are generally asymptomatic (50–90% incidental finding). Vague abdominal pain (10–15%) due to mass effect may be present. Laboratory tests for liver function are generally normal. Pathologically FNH is usually a solitary (80%), subcapsular, and nodular homogeneous mass [33]. 

FNHs could be subdivided into FNH with sinusoidal dilatation (most of the so-called telangiectatic FNH are now recognized as inflammatory HCA) and FNH with cytological atypia. On cut section in the majority of large FNHs, pathological features are fibrous septa and cellular areas of hepatic proliferation. Hepatocytes rarely may contain fats, triglycerides, and glycogens. Lesions more than 5 cm frequently show a central fibrous scar which consists of fibrous connective tissue, cholangiocellular proliferation, and malformed vessels (arteries, capillaries, and veins). The capsule is seldom present and margins are usually sharp. Unlike HCA, haemorrhage and necrosis are exceptional within the lesion. No malignant degeneration of FNH has been observed [34].

Surgical resection is suggested only in large symptomatic lesions.

**Figure 7:**
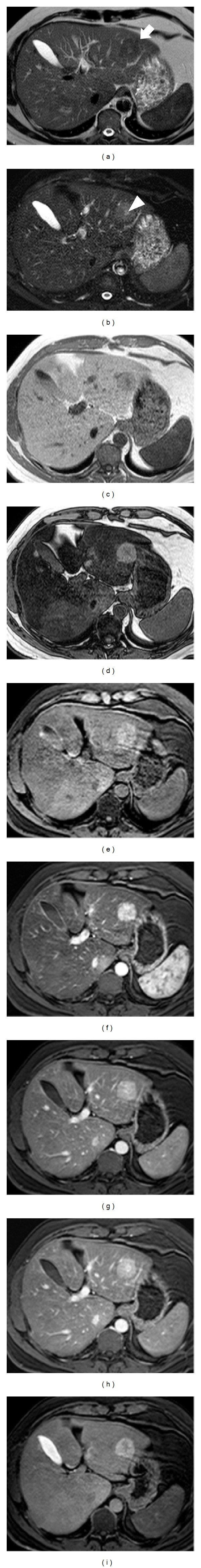
Focal nodular hyperplasia in fatty liver. (a-b) Axial T2w images without (a) and with (b) fat suppression. In S II slightly hypointense (a) and hyperintense (b) lesions (arrow) with hyperintense central scar (arrowhead). (c-d) Due to significant steatosis of hepatic parenchyma the lesion is hypo- and hyperintense in T1w in- and out-phased images. (e–h) In dynamic evaluation after Gd-BOPTA administration the nodule appears significantly hypervascular in arterial phase (f) and tends to be hyperintense in portal and equilibrium phases. Typically central scar becomes hyperintense in equilibrium phase. (i) Hepatobiliary phase image after 1 hour. The lesion is hyperintense except central scar which is hypointense with stellate aspect.

On MR imaging, classic FNH appears as homogeneously isointense or slightly hyperintense on T2w images and isointense or slightly hypointense on T1w images before contrast medium administration. Typical behaviours during dynamic study are marked and homogeneous enhancement during the arterial phase, rapid wash out during the portal phase, and isointensity (with the exception of the scar) during the equilibrium phase. In hepatobiliary phase FNH is isointense or slightly hyperintense on T1w images. In contrast, on delayed liver specific phase images after Gd-BOPTA and Gd-EOB-DTPA administration, the common appearance of HCAs is hypointensity of the solid, nonhemorrhagic components of the lesions, with the exception of the inflammatory subtype. This one is the main feature that differentiates FNHs from HCAs lesions. When present, in FNH a typical scar appears as hyperintense or hypointense stellate area, respectively, on T2 and T1-weighted images; it is hypointense during the arterial and portal-venous phases and slightly hyperintense during the equilibrium phase (Figure 7) [35–37].

### 4.2. Large Regenerative Hyperplasia

Large regenerative hyperplasia (LRH) is another generally asymptomatic rare condition (0.5–2.5% on autopsy series) characterized by diffuse micronodular transformation of the hepatic parenchyma, without fibrous septa between nodules. Disorders in the hepatic microcirculation (chronic ischemia) with hyperplastic parenchyma response seem to be the primary cause of LRH. Myeloproliferative and lymphoproliferative disorders, chronic vascular and rheumatologic syndromes, and some drugs used are related to LRH. Adults (mean age 50), without gender predilection known, are more affected by LRH, rarely reported in childhood (e.g., in congenital absence of portal vein). Acute abdominal pain occurs in case of rupture of a large subcapsular nodule with hemoperitoneum. If central scar is present nodules can be indistinguishable from FNH. Portal vein obstruction and secondary hepatic arterial dilatation can be observed. In case of splenomegaly and increased flow in portal vein, several nodules can be observed with patent portal branches. When thrombosis of a large portal branch occurs, remnant portal flow is redirected in the perihilum parenchyma with large regenerative nodules (of several cm) near the large portal tracts and small nodules with atrophy in the peripheral parenchyma. No malignant degeneration of LRH has been observed [38, 39]. 

Prognosis depends on underlying disease. Treatment is indicated in progressive hepatic failure related to underlying disease.

**Figure 8:**
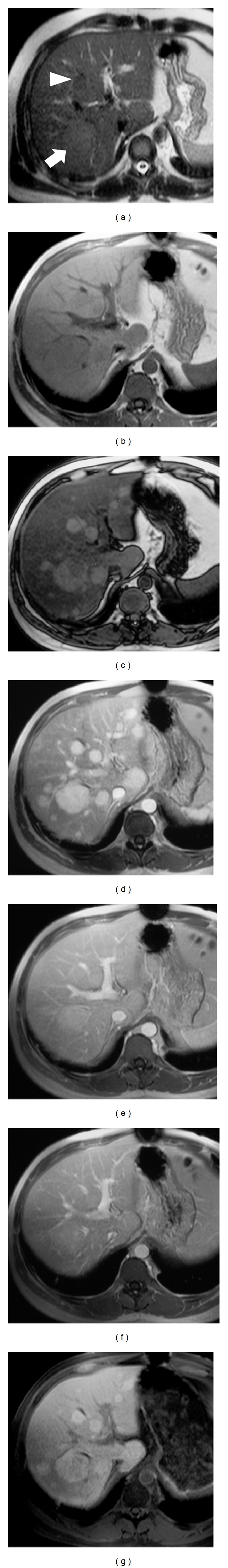
Large regenerative hyperplasia. (a) Axial T2w image. In S VII slightly hyperintense nodule (arrow); in SIV moderate hypointense lesion (arrowhead). (b-c) T1w in- and out-phased images. Due to significant steatosis of hepatic parenchyma after chemotherapy, many other hypointense lesions appear in out-phased sequence. (d–f) In dynamic evaluation after Gd-BOPTA administration the nodules appear significantly hypervascular in arterial phase (d) and tend to be isointense in portal and equilibrium phases. (g) Hepatobiliary phase image after 1 hour. Nodules are hyperintense.

On unenhanced T1w MR images LRH is generally almost isointense or slightly hyperintense compared to the surrounding liver parenchyma while on unenhanced T2w images the nodule appears iso- or slightly hypointense. A pheripheral hypointense rim is often visible in large lesions on T1- and T2-weighted images. On dynamic MR imaging, LRH is usually hyperintense in the arterial phase and iso- or slightly hyperintense in the portal-venous and equilibrium phases. In the delayed, liver specific phase after Gd-BOPTA or GD-EOB-DTPA the lesion appears isointense or hyperintense because it consists of benign hepatocytes with abnormal biliary system drainage (Figure 8) [40, 41].

The main diagnostic dilemma is between LRH and inflammatory HCA; the key elements to achieve the differential diagnosis are the different signal on T2 images (isointense or slightly hypointense in LRH, hyperintense in HCA); the dissimilar contrast behaviour in dynamic study.

### 4.3. Hepatocellular Carcinoma in Noncirrhotic Patients and Fibrolamellar Hepatocellular Carcinoma

HCC in noncirrhotic patients and fibrolamellar hepatocellular carcinoma (FL-HCC) are rare malignant primary liver tumors which arise in young healthy patients of both sexes (M : F = 1 : 1). 

Hepatomegaly, malaise, pain in right upper quadrant, fever, and/or weight loss may be present. The lesion becomes symptomatic only when the mass reaches very big size and compresses adjacent structures or invades vascular or biliary vessels (rarely jaundice can reveal the presence of tumour).

Both tumors can present with metastatic disease (lymph nodes and lungs are the most frequent sites). Alpha-fetoprotein is usually elevated in conventional HCC but often normal in FL-HCC.

Generally there is a large (average mean size >10 cm), single (80–90%), well-demarcated, lobulated, noncapsulated mass (incomplete capsule in 1/3 of cases); in 20% of cases it can be pedunculated. 

In FL-HCC coarse calcifications had been depicted in more than 50% of lesions and, at cut section, lobular arrangement fibrous septa with radial disposition and central scar are seen in 60–70% of cases; abdominal lymphadenopathy had been detected in >60% of patients. 

Central scar, fibrosis, and calcification are rare in conventional HCC whereas necrosis hemorrhage and intratumoral fat are much more common than in FL-HCC.

The surgical resectability is high (50%); multiple or too large tumors can be treated with liver transplantation. FL-HCC is frequently recurrent, but 5-year survival is about 50–60%.

**Figure 9:**
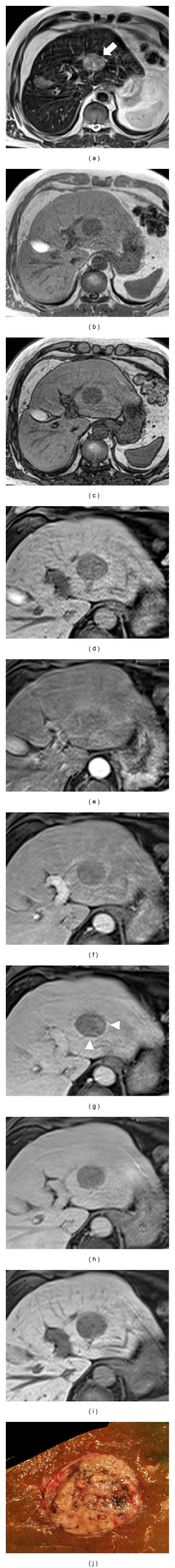
HCC in noncirrhotic liver. (a) Axial T2w image. In S II-S III slightly hyperintense well delineated lesion (arrow). (b-c) In T1w in- and out-phased images of the lesion appears hypointense. (d–h) In dynamic evaluation after Gd-EOB-DTPA administration the nodule appears slightly hypervascular in arterial phase (e); it shows rapid and progressive wash out in portal and equilibrium phases. Note the presence of a pseudocapsule (arrowhead) in equilibrium phase. (i) Hepatobiliary phase image after 20′. The lesion appears hypointense. (j) Pathologic specimen confirms the presence of HCC nodule in normal liver.

On MR imaging, HCC in non-cirrhotic patients depends largely on tumor size that is generally large because the lesion is not detected in early stage. Generally, most HCCs, because of their hypervascular nature, are hyperintense compared to the liver in the arterial phase and hypointense in the portal-venous and equilibrium phases. Irregular mosaic-like or pheripheral enhancement is usually seen in large neoplasms, depending on the internal architecture. In the delayed liver-specific phases after Gd-BOPTA or Gd-EOB-DTPA well-differentiated and moderately differentiated HCCs may show in a small percentage of cases superior signal enhancement ratios to poorly differentiated HCCs (Figure 9).

Differential diagnosis between HCC and HCA, subtypes inflammatory and *β*-catenin, is very difficult: both adenomas may be heterogeneously hyperintense on T2-weighted images and hypointense on T1-weighted sequences; they may show intense and heterogeneous enhancement in the arterial phase of dynamic study, subsequent wash out, and/or hypointense signal in hepatobiliary phase. The larger component of fat is more typical for HNF-1*α*-mutated HCA.

**Figure 10:**
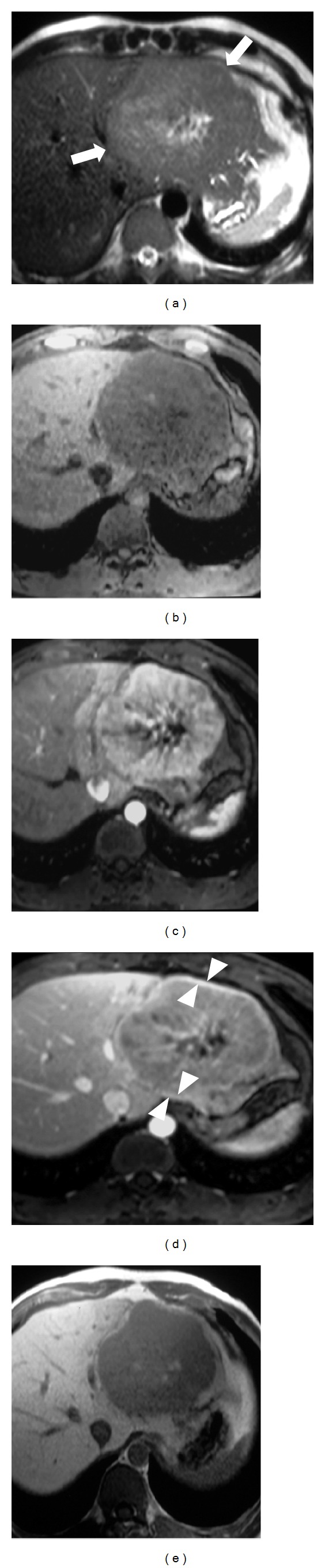
Fibrolamellar HCC. (a) Axial T2w image. In left lobe slightly well delineated mass (arrows) with heterogeneous central area. (b–d) In dynamic evaluation after Gd-BOPTA the lesion appears markedly heterogeneous hypervascular in arterial phase (c); it shows rapid and progressive wash out in portal phase. The central area is hypointense. Note complete thick pseudocapsule at the periphery (arrowhead). (e) In hepatobiliary phase image the lesion is hypointense; the central fibrotic component retains contrast agent.

FL-HCC is usually either hypointense or, rarely, isointense compared to the liver on T1-weighted images. On T2-weighted images, 90% of the lesions are hyperintense and the remaining 10% are isointense. The purely fibrous nature of the scar means that it is hypointense on both T1- and T2-weighted images. FL-HCC becomes heterogeneously hyperintense during the arterial phase after the administration of gadolinium and appears as isointense or slightly hypointense during the portal-venous and equilibrium phase. The central scar shows minimal or no enhancement in arterial and portal-venous phase images but may sometimes show persistent enhancement in equilibrium phase. In hepatobiliary phases, FL-HCC usually appears as heterogeneously hypointense with areas of low signal intensity due to necrosis or, less frequently, haemorrhage (Figure 10) [42–44].

Besides the key elements already described for HCC in non cirrhotic patients, FL-HCC may be differentiated from HCA for areas of fibrotic tissue which are hypointense on T1 and T2w images and hyperintense in the delayed phases of the dynamic studies. Signs of malignancy (vascular and biliary invasion) present in HCC and FL-HCC are other helpful elements in differential diagnosis.

### 4.4. Cholangiocarcinoma

Cholangiocarcinoma (CC) is the primary malignancy arising from the bile duct epithelium and it is the second most common liver malignancy after HCC.

Incidence of CC among primary liver tumor ranges from 5 to 30%, with an average age of 50–60 years, seen slightly more often in men (M : F = 3 : 2). Risk factors are primary sclerosing cholangitis, bile stasis, recurrent cholangitis, infections with *Clonorchis sinensis* and *Opisthorchis viverrini*, hepatolithiasis, congenital bile ducts anomalies, familiar polyposis, Thorotrast deposition, and Caroli's syndrome. Recent findings indicate that HCV-HBV infection conferred a more than twofold elevated risk of ICC.

According to the site of origin CC can be classified into two types as follows: intrahepatic or peripheral (PCC) and extra-hepatic.

PCC presents as large mass because tumor does not cause clinical symptoms in early stages. The initial symptoms are abdominal pain, malaise, anorexia, weight loss, fever, palpable mass, and jaundice (rare).

The peripheral mass appears as a large white-grey lesion characterized by fibrosis (fibrotic core) and associated with capsular retraction; calcifications are rare. Sometimes there is concomitant dilatation of adjacent bile ducts and atrophy of corresponding liver segments.

Lymph node involvement is present in 60–70% of PCC.

Metastatic spread is common: lung, bone, pancreas, adrenals, kidney, spleen, and peritoneum.

According to the Liver Cancer Study Group of Japan (LCSGJ) and based on the gross appearance ICC can be categorized in three patterns of growth that can be present alone or in to combination: mass-forming type (MF), intraductal growth (IG) type, and periductal infiltrating type (PI). The prognostic significance of such classification has been confirmed in several clinical studies [45, 46].

Curative resection is the most effective treatment and the only therapy associated with prolonged disease-free survival; the rate of radical resection is extremely variable in the literature, ranging between 30% and 80%. Prognosis of ICC is poor due to late presentation and limited resectability. Five-year survival is less than 30% of surgically treated patients.

On MR imaging PCC is either isointense or hypointense relative to the normal liver on T1w MR images but may range from mildly to markedly hyperintense on T2w images. The signal intensity of the tumour is variable and depends on the amount of mucinous material, fibrous tissue, haemorrhage, and necrosis within the lesion. 

**Figure 11:**
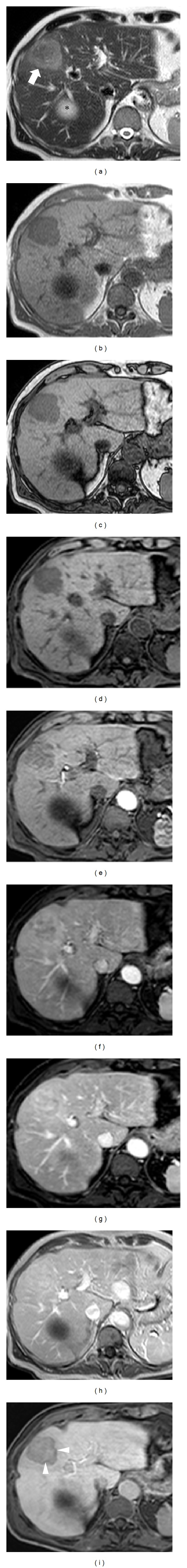
Peripheral CCC. (a) Axial T2w image. In S IV slightly heterogeneous hyperintense lobulated lesion (arrow); cyst (asterisk). (b-c) T1w in- and out-phased images. The lesion appears hypointense. (d–h) In dynamic evaluation after GD-BOPTA administration the nodule appears slightly heterogeneous hypervascular in arterial phase (e) with progressive enhancement in portal and equilibrium phases. After 5′ (h) note persistent enhancement and peripheral wash out. (i) Epatobiliary phase image after 1 h. The lesion appears hypointense, showing central pooling and more evident peripheral wash out (arrowheads).

On dynamic study after injection of Gd-BOPTA or Gd-EOB-DTPA, minimal or moderate incomplete enhancement is seen at the periphery on early images, whereas progressive central contrast enhancement is seen on later images. Generally, on delayed phase images lesions show peripheral hypointensity and central iso- or hyperintensity due to central pooling of contrast medium within central desmoplastic reaction. Satellite nodules are seen in about 10–20% of PCC cases and are chiefly responsible for the poor prognosis of this tumour (Figure 11) [47, 48]. The above three patterns are the key elements for the differential diagnosis with HCA.

### 4.5. Primary Lymphoma

Primary lymphoma (PL) of the liver (confined to the liver without involvement of lymph nodes or spleen or bone marrow) is very rare (0.4% of extranodal non-Hodgkin's lymphomas and 0.2% of all non-Hodgkin's lymphomas). PL can arise at every age but it is more frequent in childhood-adolescence or in middle-old age (M : F = 4 : 1). It can be associated with Epstein-Barr infection (EBV), and more frequently in patients with chronic hepatitis or cirrhosis (by HBV and/or HCV infections), with autoimmune disorders or with AIDS manifestations; it can also be associated with immunosuppression in transplanted patients. Liver involvement (50% of cases) in haematological diseases (secondary liver lymphoma) is more common.

Hepatomegaly, presence of hepatic mass or masses, and pain in upper right quadrant are the most frequent signs and symptoms in primary lymphoma. Fever, weight loss, and perspiration are associated in 50% of cases.

PL can present as multiple, small nodular lesions (well-differentiated B-cell lymphoma) or as diffuse infiltration of hepatic parenchyma (undifferentiated subtypes); in some patients with Hodgkin's lymphoma peliosis hepatis can be associated. 

The outcome, with appropriate treatment, is favourable. Surgical resection followed by chemotherapy and/or radiation gives the best prognosis in primary lymphoma [49, 50]. 

**Figure 12:**
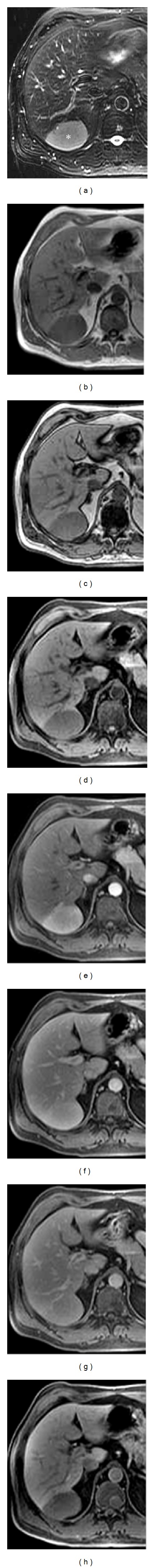
Primary hepatic NH lymphoma. (a) On T2w image well-defined homogeneous moderate hyperintense nodule (asterisk) in S VII. (b-c) T1w in- and out-phased images. The lesion appears homogeneously moderate hypointense on T1w sequences. (d–g) On contrast enhanced MRI the mass shows homogeneous hypervascular enhancement in arterial phase (e) and becomes isointense to liver parenchyma on portal and late dynamic phases. (h) In epatobiliary phase the nodule is homogeneously hypointense.

On MR imaging, PL is generally seen as homogenously/heterogeneously hypointense compared to the normal parenchyma on unenhanced T1-weighted images and hyperintense on T2-weigthed images. Dynamic imaging after Gd-BOPTA or Gd-EOB-DTPA typically reveals a hypointense appearance on arterial phase images, followed by homogeneous, delayed enhancement on portal-venous phase images, and isointensity on equilibrium phase images. However, some hypervascular PLs are described. In most cases, PL is hypointense in the delayed hepatobiliary phases (Figure 12) [51]. 

The equivocal behavior of PL does not offer important elements for differential diagnosis with HCA. However, HNF-1*α*-mutated HCA may be differentiated for the fatty intralesional component which determines signal patterns on plain MR and dynamic study.

### 4.6. Metastases

Metastases are the most common cause of malignant focal liver lesions (18–20 times more frequent than primary malignant tumors). At autopsy hepatic metastases occur in 30 to 55% of patients dying from malignant disease. Liver metastases originate predominantly from primary tumors localized in gastrointestinal tract (colon, stomach, and pancreas in decreasing order of frequency), by hematogenous spread, via portal vein. Other frequent secondary lesions are from breast and lung cancers (but also endocrine/neuro-endocrine tumors and melanoma): primary neoplasms probably give metastases by hematogenous spread, via the arterial blood supply to the liver. Lymphogenous spread occurs along bile ducts from near organs (pancreas) or from haematological diseases (lymphoma, leukaemia). Direct invasion from nearest organs (gallbladder or bile duct cancer, pancreatic neoplasms) can also be the cause of liver metastases. Liver involvement by intra-abdominal dissemination can also be observed (ovarian cancer) [52].

Most patients with metastases to the liver present with symptoms related only to the primary tumor; the asymptomatic hepatic involvement is discovered in the course of clinical evaluation. Sometimes there are no specific symptoms: weakness, weight loss, fever, and loss of appetite. Rarely, hepatomegaly, hepatic mass, or pain in upper right quadrant can be the first symptoms of liver involvement mostly when lesion/s are huge and multiple.

Metastases can vary in size, number, consistency, uniformity of growth, vascularity, and stromal response. 

The outcome depends not only on primitive tumor, but even on number, size, and tissue component of metastases. If resectable, hepatic metastases have 20–40% survival rate at 5 years (colon cancer metastases).

The differential diagnostic dilemma between HCA and metastases is mainly in the case of hypervascular, solitary lesion in patients with unknown primary cancer.

**Figure 13:**
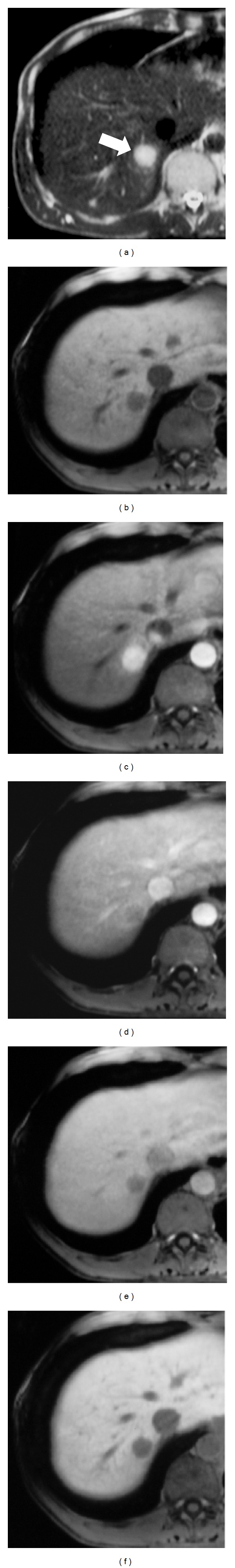
Neuroendocrine hepatic metastasis. (a) On T2w image well-defined homogeneous markedly hyperintense nodule (arrow) in S VII. (b–e) On precontrast T1w fat sat images the lesion is hypointense (b). On contrast enhanced MRI the nodule shows homogeneous evident enhancement in arterial phase (c) with rapid wash out in portal phase, more evident in equilibrium phase. (f) In epatobiliary phase, 10' after GD-EOB-DTPA, the nodule is hypointense.

Generally, on unenhanced T1w images metastases have low signal intensity compared to the surrounding parenchyma. On T2w sequences the lesions demonstrate high signal intensity, although the signal is generally lower than that typically observed in hemangiomas. Hypervascular metastases tend to reveal strong transient enhancement in the arterial phase followed by hypointensity in the portal and equilibrium phases; they appear hypointense in hepatobiliary phase (Figure 13) [41, 53, 54].

The most important elements to achieve the differential diagnosis between HCA and each type of hypervascular lesions in non cirrhotic patients are summarized in Table 1.

## 5. Conclusions

According to recent studies HCAs are currently categorized in to four distinct genetic and pathologic subtypes: inflammatory hepatocellular adenomas, hepatocyte-nuclear-factor-1-alpha (HNF-1*α*-mutated) hepatocellular adenomas, *β*-catenin-mutated hepatocellular adenomas, and unclassified adenomas. This classification has definitive management implications. MR imaging plays an important role in diagnosis and characterization, particularly in inflammatory and steatotic subtypes, as well as in identification of complications and surveillance.

Image-guided biopsy or surgical resection with histopathologic and immunohistochemical analysis is necessary for complete characterization of HCAs and in some differential diagnostic dilemmas. 

## Figures and Tables

**Table 1:** Hypervascular lesions in noncirrhotic patients. The most important elements to achieve the differential diagnosis.

MRI features											
	Risk factor and clinical setting	T2w	T1w in	T1w out	DWI	Pre-c	ArtP	PVP	EqP	HBP	Specific pattern
Inflammatory adenoma		Hyper	Hypo	Hypo	Restriction	Hypo	Hyper +	Hyper	Hyper/iso	Hypo/hyper	Signal heterogeneity, hypervascularity, intralesional hemorrhage
HNF-1*α*-mutated adenoma	Obesity, steatosis, diabetes, glycogenesis, exogenous steroids	Iso/hyper	Hyper	Hypo	No restriction	Iso/hypo	Hyper	Hypo	Hypo	Hypo	Fatty intralesional component
*β*-catenin-mutated adenoma		Hyper	Hypo	Hypo	Restriction	Hypo	Hyper +	Hyper	Hyper/iso	Hypo/hyper	Intralesional hemorrhage, necrosis
Unclassified adenoma											Nonspecific patterns reported
Focal nodular hyperplasia	Exogenous steroids	Iso	Iso	Iso	No restriction	Iso	Hyper +++	Iso	Iso	Iso/hyper	Signal homogeneity, hypervascularity, scar, iso/hyperintensity on HbP
Large regenerative nodule	Myeloproliferative and lymphoproliferative disorders, chronic vascular and rheumatologic syndromes	Iso	Iso	Iso	No restriction	Iso	Hyper ++	Iso	Iso	Iso/hyper	Multiple lesions signal homogeneity, hypervascularity, scar, isohyperintensity on HbP
HCC in noncirrhotic liver and FL-HCC	Exogenous steroidenvironmental and dietary factors (aflatoxins and nitrosamine)	Hyper	Hypo	Hypo	Restriction	Hypo	Hyper ++	Hypo	Hypo	Hypo	Signal heterogeneity, intralesional hemorrhage, signs of malignancy
Cholangiocarcinoma	Viral hepatic infection	Hyper	Hypo	Hypo	Restriction	Hypo	Hyper	Hypo	Hypo/iso	Hypo/hyper	Peripheral enhancement, peripheral washout, central pooling, signs of malignancy
Primary lymphoma	Epstein-Barr infection (EBV), HBV and/or HCV infections	Iso/hyper	Hypo	Hypo	Restriction	Hypo	Hyper	Hypo	Hypo	Hypo	Variable pattern, small lesions hypervascular, significant restriction
Metastases	Primary tumor, metastatic syndrome	Hyper	Hypo	Hypo	Restriction	Hypo	Hyper ++	Iso/hypo	Hypo	Hypo	Multiple lesions hypervascularity, halo signs signs of malignancy

## References

[1] La Vecchia C, Tavani A (2006). Female hormones and benign liver tumours. *Digestive and Liver Disease*.

[2] Prentice RL (1991). Epidemiologic data on exogenous hormones and hepatocellular carcinoma and selected other cancers. *Preventive Medicine*.

[3] Labrune P, Trioche P, Duvaltier I, Chevalier P, Odièvre M (1997). Hepatocellular adenomas in glycogen storage disease type I and III: a series of 43 patients and review of the literature. *Journal of Pediatric Gastroenterology and Nutrition*.

[4] Giannitrapani L, Soresi M, La Spada E, Cervello M, D’Alessandro N, Montalto G (2006). Sex hormones and risk of liver tumor. *Annals of the New York Academy of Sciences*.

[5] Cappell MS (2008). Hepatic disorders severely affected by pregnancy: medical and obstetric management. *Medical Clinics of North America*.

[6] Greaves WOC, Bhattacharya B (2008). Hepatic adenomatosis. *Archives of Pathology and Laboratory Medicine*.

[7] Yoshidome H, McMasters KM, Edwards MJ (1999). Management issues regarding hepatic adenomatosis. *American Surgeon*.

[8] Colli A, Fraquelli M, Massironi S, Colucci A, Paggi S, Conte D (2007). Elective surgery for benign liver tumours. *Cochrane Database of Systematic Reviews*.

[9] Bioulac-Sage P, Rebouissou S, Thomas C (2007). Hepatocellular adenoma subtype classification using molecular markers and immunohistochemistry. *Hepatology*.

[10] Bioulac-Sage P, Balabaud C, Zucman-Rossi J (2010). Subtype classification of hepatocellular adenoma. *Digestive Surgery*.

[11] Bioulac-Sage P, Laumonier H, Couchy G (2009). Hepatocellular adenoma management and phenotypic classification: the Bordeaux experience. *Hepatology*.

[12] Zucman-Rossi J, Jeannot E, Van Nhieu JT (2006). Genotype-phenotype correlation in hepatocellular adenoma: new classification and relationship with HCC. *Hepatology*.

[13] Lewin M, Handra-Luca A, Arrivé L (2006). Liver adenomatosis: classification of MR imaging features and comparison with pathologic findings. *Radiology*.

[14] Laumonier H, Bioulac-Sage P, Laurent C, Zucman-Rossi J, Balabaud C, Trillaud H (2008). Hepatocellular adenomas: magnetic resonance imaging features as a function of molecular pathological classification. *Hepatology*.

[15] Katabathina VS, Menias CO, Shanbhogue AKP (2011). Genetics and imaging of hepatocellular adenomas: 2011 update. *Radiographics*.

[16] Rohrer M, Bauer H, Mintorovitch J, Requardt M, Weinmann HJ (2005). Comparison of magnetic properties of MRI contrast media solutions at different magnetic field strengths. *Investigative Radiology*.

[17] Kim HJ, Kim BS, Kim MJ (2012). Enhancement of the liver and pancreas in the hepatic arterial dominant phase: comparison of hepatocyte-specific MRI contrast agents, gadoxetic acid andgadobenate dimeglumine, on 3 and 1.5 tesla MRI in the same patient. *Journal of Magnetic Resonance Imaging*.

[18] Feuerlein S, Gupta RT, Boll DT, Merkle EM (2011). Hepatocellular MR contrast agents: enhancement characteristics of liver parenchyma and portal vein after administration of gadoxetic acid in comparison to gadobenate dimeglumine. *European Journal of Radiology*.

[19] Lee MS, Lee JY, Kim SH (2011). Gadoxetic acid disodium-enhanced magnetic resonance imaging for biliary and vascular evaluations in preoperative living liver donors: comparison with gadobenate dimeglumine-enhanced MRI. *Journal of Magnetic Resonance Imaging*.

[20] Filippone A, Blakeborough A, Breuer J (2010). Enhancement of liver parenchyma after injection of hepatocyte-specific MRI contrast media: a comparison of gadoxetic acid and gadobenate dimeglumine. *Journal of Magnetic Resonance Imaging*.

[21] Denecke T, Steffen IG, Agarwal S (2012). Appearance of hepatocellular adenomas on gadoxetic acid-enhanced MRI. *European Radiology*.

[22] Bioulac-Sage P, Laumonier H, Laurent C, Zucman-Rossi J, Balabaud C (2008). Hepatocellular adenoma: what is new in 2008. *Hepatology International*.

[23] Paradis V, Champault A, Ronot M (2007). Telangiectatic adenoma: an entity associated with increased body mass index and inflammation. *Hepatology*.

[24] Bioulac-Sage P, Rebouissou S, Sa Cunha A (2005). Clinical, morphologic, and molecular features defining so-called telangiectatic focal nodular hyperplasias of the liver. *Gastroenterology*.

[25] Mohajer K, Frydrychowicz A, Robbins JB (2012). Characterization of hepatic adenoma and focal nodular hyperplasia with gadoxetic acid. *Journal of Magnetic Resonance Imaging*.

[26] Grazioli L, Bondioni MP, Haradome H (2012). Hepatocellular adenoma and focal nodular hyperplasia: value of gadoxetic acid-enhanced MR imaging in differential diagnosis. *Radiology*.

[27] Bioulac-Sage P, Frédéric Blanc J, Rebouissou S, Balabaud C, Zucman-Rossi J (2007). Genotype phenotype classification of hepatocellular adenoma. *World Journal of Gastroenterology*.

[28] Bluteau O, Jeannot E, Bioulac-Sage P (2002). Bi-allelic inactivation of TCF1 in hepatic adenomas. *Nature Genetics*.

[29] Kanematsu M, Goshima S, Watanabe H (2012). Detection and characterization of focal hepatic lesions with diffusion-weighted MR imaging: a pictorial review. *Abdominal Imaging*.

[30] Miller FH, Hammond N, Siddiqi AJ (2010). Utility of diffusion-weighted MRI in distinguishing benign and malignant hepatic lesions. *Journal of Magnetic Resonance Imaging*.

[31] Chen YW, Jeng YM, Yeh SH, Chen PJ (2002). p53 gene and Wnt signaling in benign neoplasms: *β*-catenin mutations in hepatic adenoma but not in focal nodular hyperplasia. *Hepatology*.

[32] Farges O, Ferreira N, Dokmak S, Belghiti J, Bedossa P, Paradis V (2011). Changing trends in malignant transformation of hepatocellular adenoma. *Gut*.

[33] Maillette De Buy Wenniger L, Terpstra V, Beuers U (2010). Focal nodular hyperplasia and hepatic adenoma: epidemiology and pathology. *Digestive Surgery*.

[34] Kreitner KF, Thelen M, Schild H (1987). Epidemiologische und klinische aspekte der fokalnodularen hyperplasie der leber. *Deutsche Medizinische Wochenschrift*.

[35] Grieser C, Steffen IG, Seehofer D (2012). Histopathologically confirmed focal nodular hyperplasia of the liver: Gadoxetic acid-enhanced MRI characteristics. *Journal of Magnetic Resonance Imaging*.

[36] Vilgrain V (2006). Focal nodular hyperplasia. *European Journal of Radiology*.

[37] Hussain SM, Terkivatan T, Zondervan PE (2004). Focal nodular hyperplasia: findings at state-of-the-art MR imaging, US, CT, and pathologic analysis. *Radiographics*.

[38] Wanless IR (1990). Micronodular transformation (nodular regenerative hyperplasia) of the liver: a report of 64 cases among 2,500 autopsies and a new classification of benign hepatocellular nodules. *Hepatology*.

[39] Wanless IR (1996). Nodular regenerative hyperplasia, dysplasia, and hepatocellular carcinoma. *American Journal of Gastroenterology*.

[40] Ames JT, Federle MP, Chopra K (2009). Distinguishing clinical and imaging features of nodular regenerative hyperplasia and large regenerative nodules of the liver. *Clinical Radiology*.

[41] Morana G, Grazioli L, Kirchin MA (2011). Solid hypervascular liver lesions: accurate identification of true benign lesions on enhanced dynamic and hepatobiliary phase magnetic resonance imaging after gadobenate dimeglumine administration. *Investigative Radiology*.

[42] Ichikawa T, Federle MP, Grazioli L, Madariaga J, Nalesnik M, Marsh W (1999). Fibrolamellar hepatocellular carcinoma: imaging and pathologic findings in 31 recent cases. *Radiology*.

[43] Ichikawa T, Federle MP, Grazioli L, Marsh W (2000). Fibrolamellar hepatocellular carcinoma: pre- and posttherapy evaluation with CT and MR imaging. *Radiology*.

[44] Campos JT, Sirlin CB, Choi JY (2012). Focal hepatic lesions in Gd-GD-EOB-DTPA enhanced MRI: the atlas. *Insights Imaging*.

[45] Sempoux C, Jibara G, Ward SC (2011). Intrahepatic cholangiocarcinoma: new insights in pathology. *Seminars in Liver Disease*.

[46] Guedj N, Bedossa P, Paradis V (2010). Anatomopathologie des cholangiocarcinomes. *Annales de Pathologie*.

[47] Maetani Y, Itoh K, Watanabe C (2001). MR imaging of intrahepatic cholangiocarcinoma with pathologic correlation. *American Journal of Roentgenology*.

[48] Manfredi R, Barbaro B, Masselli G, Vecchioli A, Marano P (2004). Magnetic resonance imaging of cholangiocarcinoma. *Seminars in Liver Disease*.

[49] Zafrani ES, Gaulard P (1993). Primary lymphoma of the liver. *Liver*.

[50] Ryan J, Straus DJ, Lange C (1988). Primary lymphoma of the liver. *Cancer*.

[51] Soyer P, Van Beers B, Grandin C, Pringot J, Levesque M (1993). Primary lymphoma of the liver: MR findings. *European Journal of Radiology*.

[52] Ihse I, Persson B, Tibblin S (1995). Neuroendocrine metastases of the liver. *World Journal of Surgery*.

[53] Hayashi D, Tkacz JN, Hammond S (2011). Gastroenteropancreatic neuroendocrine tumors: multimodality imaging features with pathological correlation. *Japanese Journal of Radiology*.

[54] Namasivayam S, Martin DR, Saini S (2007). Imaging of liver metastases: MRI. *Cancer Imaging*.

